# Making Sense of Real-Time Functional Magnetic Resonance Imaging (rtfMRI) and rtfMRI Neurofeedback

**DOI:** 10.1093/ijnp/pyv020

**Published:** 2015-04-08

**Authors:** Annette B. Brühl

**Affiliations:** University of Cambridge, Behavioural and Clinical Neuroscience Institute and Department of Psychiatry, Downing site, Cambridge, United Kingdom; Department of Psychiatry, Psychotherapy and Psychosomatics, University Hospital of Psychiatry Zurich, Zurich, Switzerland.

**Keywords:** Brain, control, regulation, mental disorders, neuroimaging.

## Abstract

This review explains the mechanism of functional magnetic resonance imaging in general and specifically introduces real-time functional magnetic resonance imaging as a method for training self-regulation of brain activity. Using real-time functional magnetic resonance imaging neurofeedback, participants can acquire control over their own brain activity. In patients with neuropsychiatric disorders, this control can potentially have therapeutic implications. In this review, the technical requirements are presented and potential applications and limitations are discussed.

## The Neurovascular Coupling as Basic Mechanism of Functional Magnetic Resonance Imaging

**Figure 1.**
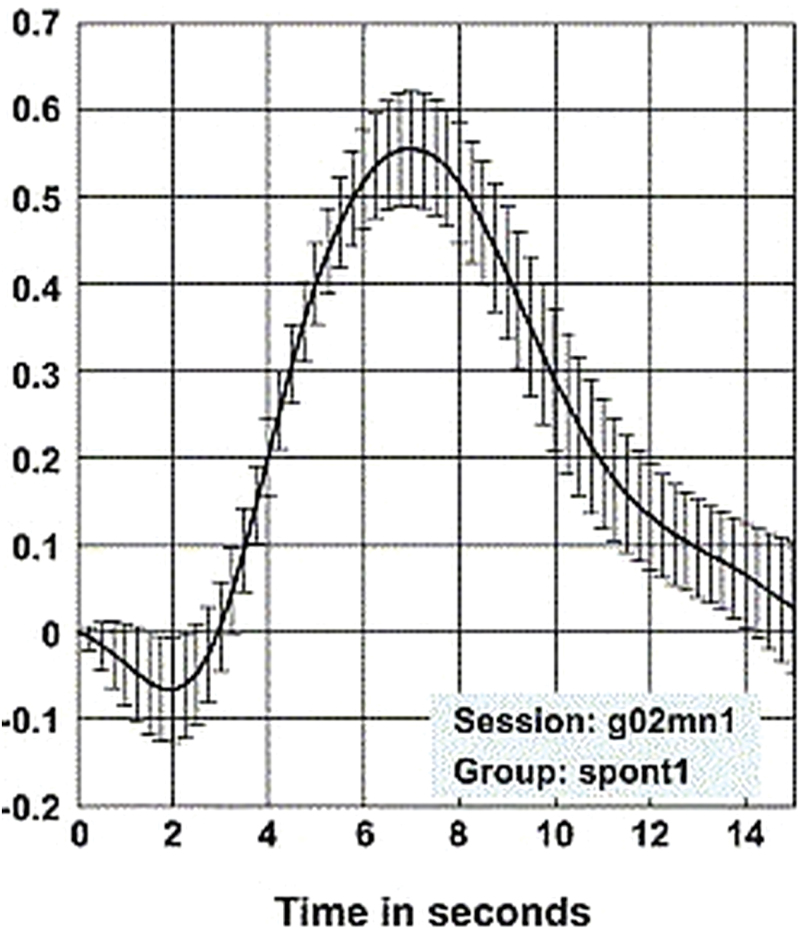
Hemodynamic blood-oxygen level dependent (BOLD) response modelled from neural responses to a short stimulus. From Logothetis and Pfeuffer, 2004, with permission.

In the early 1990s, S. Ogawa described the so-called blood-oxygen level dependent (BOLD) effect (reviewed in Kim and Ogawa, 2012), which is the basis of the signal that is used in functional magnetic resonance imaging (fMRI). The BOLD effect is based on 2 principles: first, the differential magnetization and therefore signal characteristics of oxygenated compared with desoxygenated haemoglobin, and second, the neurovascular coupling, referring to an autoregulatory process during which the increased glucose and oxygen consumption in active brain areas is overcompensated by overly increased perfusion, leaving these activated areas with a surplus of oxygenated blood or haemoglobin. This surplus is then measured as change in magnetization signal in the MRI. The time curve of this reaction (hemodynamic response function; Buckner, 1998; Logothetis et al., 2001) is rather slow: after an initial short “undershoot” due to the increased oxygen consumption in an activated area, the signal peaks after about 5 to 6 seconds and returns to baseline 12 to 15 seconds after the initial activation (Figure 1).

The signal change due to functional activation is overall rather weak compared with the noise, reaching up to 5% in primary visual cortex and usually around 0.5% to 1.5% in other brain areas compared with baseline (usually the period before the stimulation or between the stimulation blocks). Therefore, typical fMRI studies need to repeat the stimulation, for example, show a number of stimuli of the same category (such as emotional pictures), or to repeat the same processes (eg, words, movements) to reliably identify the signal amongst the noise (ie, signal fluctuations not related to external stimuli or processes). The analysis of the fMRI data is typically done after the acquisition of the data and involves removal of movement-associated artefacts, filtering, and co-registration with the individual anatomical data. Thereafter, the data are in most cases normalized, that is, brought into a standardized space grid (either the Talairach space or the MNI system), in which each brain area can be described with 3 coordinates to enable group-wise analysis and then the comparison between groups or even studies.

The strengths of fMRI compared with other techniques used to measure brain processes are: (1) the high spatial resolution (up to 1×1×1mm^3^); (2) the coverage of the whole brain (compared with EEG or MEG, which both have a lower spatial resolution and are rather limited to cortical regions/regions close to the skull, other regions can be extrapolated, but not directly measured); and (3) the noninvasiveness (compared with PET and similar techniques using injected radioactive tracers).

## What Is Real-Time fMRI (rtfMRI)?

One common definition of real-time fMRI (rtfMRI) is “any process that uses functional information from a MRI scanner where the analysis and display of the fMRI data keep pace with the data acquisition” (Sulzer et al., 2013b). With the improvement of MRI acquisition speed, data transfer techniques, and computation capacities and algorithms, it is now possible to directly transfer the raw data from the MRI scanner to a PC/laptop computer as soon as they are acquired. This computer can then process the fMRI data as described above in (near) real-time and in an incremental way (new incoming data is included into the statistical model as it is acquired (Hinds et al., 2011). There are a couple of possible applications for rtfMRI:

- Quality control during acquisition of fMRI data (reviewed in Weiskopf et al., 2007)
- Control of changes in subject’s attention and performance (reviewed in Weiskopf et al., 2007)
- Rapid pre-/intrasurgical detection of functionally important brain regions such as language dominance, motor cortex (eg, Moller et al., 2005; Schwindack et al., 2005)
- Use of the fMRI signal for neurofeedback, enabling the subject in the scanner to control its own brain activity (recent reviews: Caria et al., 2012, Weiskopf, 2012, Sulzer et al., 2013b)
- Development of adaptive cognitive tasks (eg, timing of task conditions according to intrinsic fluctuations of brain activity, Yoo et al., 2012).

Since the 1960s, EEG has been used as a source for neurofeedback, that is, acquiring volitional control over one’s own brain activity (Larsen and Sherlin, 2013). Compared with the EEG signal, which has a time resolution of milliseconds, the fMRI signal is rather slow. However, due to recent technical improvements, it is now possible to acquire fMRI data of the whole brain every 500 milliseconds (Feinberg et al., 2010). Nevertheless, most rtfMRI studies still use sampling rates of 1.5 to 2 seconds. This might sound slow, but as the BOLD effect is even slower (see above, peak 5 seconds after a 1-second stimulus), improvements in sampling rate are not the first priority in the further development of rtfMRI.

## Specific Technical Aspects of rtfMRI Experiments

Compared with classical offline analyzed fMRI experiments, rtfMRI experiments have to be more robust, as they aim to measure the signal changes with each single trial of the task. Furthermore, the signal change must be strong and robust enough already in a single subject (as compared with experiments measuring small signal differences in groups of participants, usually >15–20). In addition to using robust tasks, other acquisition parameters are typically optimized to get a good spatial resolution with a low degree of distortion and a high signal-to-noise ratio but still low acquisition rates. Some studies therefore cover not the whole brain or cut back on spatial resolution (typically 3×3×3mm^3^ or larger).

The online or real-time data analysis requires that the MRI scanner allows access to the data in real-time (without storing them internally) and in a continuous order. The data is then transferred via a fast network connection to a high performing computer unit where the analysis is done. Real-time data analysis aims at rapidly identifying and extracting artefacts, for instance due to head movements, breathing, and cardiovascular pulsation, and to then identify and analyze those signal changes that are related to the brain activation. These signals are typically computed based on conventional fMRI analysis methods such as general linear models, which are computed incrementally, sometimes using a sliding window for the baseline definition or the signal change quantification. Classical rtfMRI experiments measure BOLD-related signals from a single region of interest (ROI). Recently, more complex signals have been used based on the differential activity between 2 ROIs or a whole network of ROIs or the connectivity (synchronicity) between defined ROIs (Koush et al., 2013). The individual ROIs can be defined either anatomically based on landmarks or functionally, often by applying a so-called localizer task first. The localizer task is constructed such that it reliably (and maybe even selectively) activates the target area and enables therefore the definition of the source ROI from which then the signal in the subsequent real-time experiment can be measured. For the definition of the ROI researchers frequently use a combination of functional activation and anatomical landmarks.

## How Does rtfMRI Neurofeedback Work?

**Figure 2.**
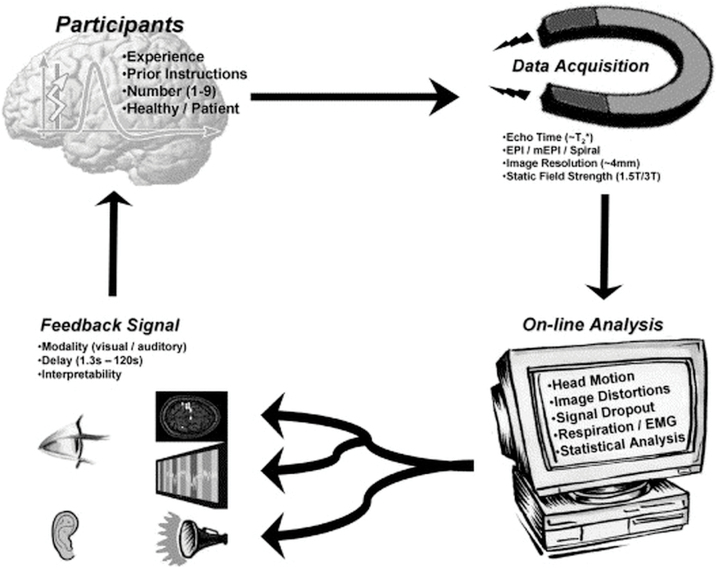
Principle and set-up of a real-time functional magnetic resonance imaging (rtfMRI) neurofeedback experiment and data-flow. From Weiskopf et al., 2004 with permission.

Neurofeedback means that an individual receives feedback based on neuronal activity which can then be influenced and regulated (Figure 2, Weiskopf et al., 2004). To be useful for an individual, the feedback must be sufficiently different from the noise (and artefacts) such that the individual can discriminate it and detect changes in the feedback signal. When planning and conducting a study, it is therefore important that the participants can understand the feedback signal and the signal changes. It is furthermore important that the participants in the study are aware of the delay of the physiological source signal due to the delay of the BOLD effect. This means that after a single visual stimulus, the neurofeedback signal can be detectable for the subject only 2 to 4 seconds later. If the subject then starts a regulatory activity, this will affect the feedback signal at least 3 to 4 seconds later, eventually (depending on specifics of brain regions and processes) even later. This delay must be explained to the participants when trying to learn to self-regulate brain activity. If participants are informed about this delay and eventually even pretrained (for instance with computer-based trainings), they can adapt their expectations and behavior to the delay.

In most situations, participants will need instructions and examples about the relationship between external stimulation or behavior and the feedback signal (eg, if it is related to language, motor activity, emotion, or visual stimuli) and will also need examples of possible strategies how to influence the signal.

Because of the specific situation in the MR scanner, the communication with the participants and presentation of stimuli and feedback is usually done visually. Studies have used various visualizations of the feedback signal. Many studies used a thermometer-like representation, where the increase or decrease of the bar (or another marker) reflects the change of brain activity (eg, Rota et al., 2009). Other studies used other visualizations such as the change of color blocks from blue (cool) to red (hot; Brühl et al., 2014), the size of a stimulus (eg, fire; deCharms et al., 2005), the intensity of a smile (as additional socially rewarding reinforcer; Mathiak et al., 2010), or a weight being lifted. Many studies have trained participants to control a brain region without additional external stimulation (ie, in an otherwise resting state), but some studies recently have used external stimuli with or against which participants were trained to regulate their brain activity. For instance, the study by Brühl et al. (2014) instructed participants to downregulate their amygdala activity while watching negative emotional pictures, which are known to activate the amygdala.

Until now, studies have shown that it is in principle possible to acquire control over (nearly) all brain regions if the signal is presented in an understandable way and if they are given at least a hint of a possible strategy how to control the brain region.

The process of learning to control a brain region is difficult and demanding. Therefore, researchers need to take into account the cognitive capacity and attention span, such that the training should be done in appropriate blocks of 5 to 20 minutes each and not exceed 30 to 60 minutes in total per session. Furthermore, offering a strategy or a couple of strategies might help participants acquiring control over a brain region more quickly and more successfully. The process of learning to control the signal derived from brain activity can be due to explicit learning, for example, when the participants are being told about the function of the brain region and therefore about regulatory strategies associated with this function. However, many studies have rather used an implicit approach by not explaining the sometimes multiple and not very tangible functions of brain areas or even networks, but instead recommending a group of strategies known to influence the brain region/network, which makes it easier for people without neuroscience knowledge and also patients with neurological or mental disorders to acquire control of the signal (for instance in depression; Linden et al., 2012).

## Current Research and Future Applications

One important challenge for the application of rtfMRI neurofeedback for any clinical or other training purposes is the transfer. Transfer here means 2 things: (1) transferring the control acquired while receiving fMRI feedback into a situation without feedback (task in fMRI without feedback, task outside the MRI without feedback), and (2) transferring the learned skill onto other tasks or processes, at best into reality and into a function where the learned skill will be helpful in everyday life.

Transfer is necessary for rtfMRI neurofeedback training to be applied in reality, because the skill should also be applicable outside the scanner without the feedback to be useful. In theory, extensive training should result in storing the newly acquired skill similar to a learned motor skill in basal ganglia and cortical circuits via neuroplastic mechanisms (Birbaumer et al., 2013). At the moment, many studies show good acquisition of control inside the scanner, but the evidence for ongoing behavioral changes in real life after the training is limited (Stoeckel et al., 2014).

Despite the increasing availability of MRI scanners, the costs of MRI scanning should be taken into account when comparing rtfMRI neurofeedback with other therapeutic techniques with known efficacy. Many studies currently focus on proving the effect of rtfMRI neurofeedback on acquiring control over a brain region or network, showing effects at the behavioral and subjective level, and optimizing protocols (eg, length of sessions, number of training sessions). However, after establishing this effect, future studies will have to focus more strongly on the transfer to prove an effect of rtfMRI neurofeedback for clinical and other use (eg, rehabilitation, training).

The development of rtfMRI for treating disorders of the brain will follow similar phases as in the development of a pharmacological treatment or another therapeutic intervention. First, in phases 0 and I, the aim is to show an overall effect in a few healthy participants, based on preclinical and pathophysiological models and theories, and also to investigate dose-response relationships. Then in phase II, studies will investigate safety and efficacy of an intervention. One large study reported on safety and side-effects of rtfMRI in a clinical population of chronic pain patients and found no specific adverse events of fMRI or rtfMRI (Hawkinson et al., 2012). So, the method in general seems to be fairly safe. However, more specific unwanted or yet unknown effects of self-regulating brain regions should be monitored. For instance, one study found in patients suffering from schizophrenia that training to upregulate the anterior insula using rtfMRI resulted in an increased detection of disgust in facial expressions (which had not been intended; Ruiz et al., 2013). To investigate efficacy in this phase of potential clinical application, studies will also compare with placebo conditions such as feedback from other brain regions or feedback using meaningless signal sources to also control for placebo effects. Studies in this phase should also strongly focus on transfer into reality and effect on clinical parameters before, and then in larger phase III studies the clinical effects can be tested and confirmed.

Potential clinical applications for such regulation could be found in multiple disorders of the brain, ranging from stroke to Parkinson’s disease to addiction and other mental disorders where brain circuits are dysregulated. Table 1 provides an up-to-date overview of studies in patient groups and the brain regions from which feedback was given. The sample size in these patient studies (as well as in most studies in healthy volunteers) was between 2 and 40.

**Table 1.** Summary of Studies Using rtfMRI Neurofeedback in Patients Suffering From Neurological and Psychiatric Disorders

**Disorder**	**Study**	**N**	**Brain Region**	**↑/↓**	**Outcome/Result**	**Disorder**
Chronic pain	deCharms et al., 2005	12 patients nf 8 HCS nf other region, 4 yoked nf	Rostral ACC	↑, ↓	Patients learned control, parallel decrease in pain intensity, no change in intensity, unpleasantness in controls	Pain localizer
Parkinson’s Disease	Subramanian et al., 2011	10 patients, 5 nf, 5 yoked feedback	Supplementary motor cortex	↑	Stronger activation in nf group, clinical improvement only in nf group	Movement localizer, motor imagery, 2 sessions (2–6 m apart), practising at home
Chronic stroke	Sitaram et al., 2012	2 patients nf, 4 HCS nf no ctr without nf	Premotor cortex	↑	Control increasing over sessions, more in patients, behavioral improvement in 1 patient and 3 HCS	Three sessions (daily), in addition TMS, behavioural task before and after training
Schizophrenia	Ruiz et al., 2013	6 patients nf, no ctr	Anterior insula	↑	Control increasing, activation in transfer run trend), increased recognition of disgust, reduced recognition of happy faces	Four sessions (daily), transfer session (no nf) 5^th^ day, after each nf training facial emotion recognition as test of transfer
MDD	Linden et al., 2012	8 nf, 8 without nf (outside scanner)	VLPFC, DLPFC, insular cortex, medial temporal lobe, OFC	↑	Control increasing, HAMD improved in nf group, no change in control group, correl w change in nf control	Four sessions (1–2 weeks apart), positive imagery/ memories localizer, varying ROIs across sessions
	Young et al., 2014	14 nf, 7 ctr (other region)	Amygdala L	↑	Control over both regions successful, clinical/mood effects stronger in amygdala group (specificity)	Happy memories as localizer, single session, transfer (without nf) comparable to last nf run
	Yuan et al., 2014	14 MDD patients nf, 27 HCS nf, 13 MDD patients ctr (other region, overlap with Young et al., 2014)	Amygdala L	↑		Resting state fMRI connectivity with amygdala before/ after nf, connectivity to pgACC normalized with nf
Smoking	Li et al., 2013	8 smokers nf, no ctr	ACC	↓	Increasing control achieved, reduced cue induced craving after training, correlating with ACC reduction	Craving as localizer, single session
	Canterberry et al., 2013	9 smokers nf, no ctr	ACC	↓	Control achieved, no additional improvement over sessions, lower craving report after nf, severity of smoking predicted nf success	Craving as localizer, three sessions
	Hanlon et al., 2013	21 nf (14 completers), no ctr	vACC, MPFC	↓/↑	Noncompleters better/quicker control, main effect of feedback on ACC regulation	Three sessions (7–10 days apart), parallel feedback from both ROIs
Psychopathy (criminal)	Sitaram et al., 2014	4 nf no ctr	Anterior insula L	↑	1/4 learned control and more aversive rating of pictures (lowest psychopathy score)	Mental imagery (neg) localizer, 1–4 sessions (daily), monetary incentive for regulation, pre/ post transfer task (emotional picture rating)
Contamination anxiety	Scheinost et al., 2013	12 nf, 11 sham	OFC	↓/↑	Clinical improvement only with nf, increased connectivity with ParCort	Two weekly sessions, contamination picture localizer,
OCD/ Contamination anxiety	Scheinost et al., 2014	5 OCD patients nf, , 10 anxious nf (overlap w. Scheinost et al. 2013)	OFC, anterior PFC	↓/↑	Clinical improvement in OCD patients with nf	1–2 sessions, OFC connectivity predicts nf success
Chronic tinnitus	Haller et al., 2010	6 nf, no ctr	Auditory cortex	↓	5/6 managed downregulation, improved symptoms in 2/6	Auditory localizer No transfer

Abbreviations: nf, neurofeedback; ACC, anterior cingulate cortex; ctr, control; DLPFC, dorsolateral PFC; HAMD, Hamilton Depression Score; HCS, healthy control subject; MDD, major depressive disorder; L, left; PFC, prefrontal cortex; pgACC, pregenual ACC, MPFC, medial PFC; OFC, orbitofrontal cortex, R, right; ↑/↓ direction of regulation, vACC, ventral ACC, ParCort parietal cortex; VLPFC, ventrolateral PFC.

The studies in patient samples are overall mostly positive, meaning that the patients managed to control the brain region from which the feedback signal was derived. However, these studies are still at the level of pilot studies, testing the feasibility of such methods in patients, and were not designed with the same rigorous design and measures as in clinical trials (eg, no placebo treatment, no comparison with gold-standard therapy, only completers reported, no intention to treat analysis, no clinical outcome measures reported, no transfer/generalization reported). Therefore, the clinical application of rtfMRI neurofeedback should still be considered to be at an experimental stage, requiring more research at the preclinical (ie, healthy participants) level, such as into optimal design of studies and tasks, optimal design of feedback signal, transfer/generalization tasks, dose-effect relationship/optimal duration, and number of sessions, etc.). This will make sure that this interesting method can be used in a responsible and evidence-based way.

rtfMRI neurofeedback will be most successful in those disorders where the pathophysiological substrate, that is, the dysfunction brain region or circuit, is well characterized. If used carefully and tested appropriately, it can be a very successful addition to known pharmacological and psychological treatments, compared by some researchers with endogenous deep-brain stimulation or even endogenous pharmacological intervention (eg, potential increase of dopaminergic tone by self-regulation of the dopaminergic midbrain; Sulzer et al., 2013a).

## Interest Statement

None.
